# Influencing factors of false lumen thrombosis in type B aortic dissection: A single-center retrospective study

**DOI:** 10.1515/med-2025-1179

**Published:** 2025-05-07

**Authors:** Qian-Hui Tang, Han Yang, Zhong Qin, Qiu-Ning Lin, Ming Hu, Xiao Qin, Jing Chen

**Affiliations:** Department of Vascular and Endovascular Surgery, The First Affiliated Hospital of Guangxi Medical University, Nanning, 530021, Guangxi, China

**Keywords:** type B aortic dissection, false lumen, thrombosis, preoperative, length

## Abstract

**Objectives:**

To evaluate the factors associated with false lumen thrombosis in preoperative type B aortic dissection (TBAD) patients.

**Methods:**

Between January 2008 and December 2017, TBAD patients were evaluated. Participants were divided into patent, partial thrombosis, and complete thrombosis groups according to the status of the false lumen. Univariate analysis and logistic regression analysis were used to identify the factors associated with false lumen thrombosis.

**Results:**

A total of 285 participants fulfilled our inclusion criteria. Patients in the complete thrombosis group were significantly older than those in the partial thrombosis and patent groups (59 ± 10 vs 53 ± 11 and 50 ± 11 years, respectively), and combined shorter dissection lengths (290 ± 101 vs 354 ± 92 and 351 ± 102 mm, respectively). The following factors were associated with partial thrombosis of the false lumen with an odds ratio (OR) <1: primary tear size (OR, 0.96; 95% confidence interval [CI], 0.93–0.99; *p* = 0.03), last tear size (OR, 0.93; 95% CI, 0.87–0.99; *p* = 0.04), and the total number of tears (OR, 0.87; 95% CI, 0.78–0.98; *p* = 0.02).

**Conclusions:**

TBAD patients older than 59 years or with aortic dissection lengths of less than 290 mm were more likely to have a thrombosed false lumen.

## 1 Introduction

Aortic dissection (AD) is a life-threatening aortic disease [1], and the International Registry of Acute Aortic Dissection reported that approximately 33% of AD cases involve type B aortic dissection (TBAD) [2]. Although thoracic endovascular aortic repair (TEVAR) is recommended to manage complicated TBAD [3], there is no consensus on the management of uncomplicated TBAD. According to the Acute Dissection Stent Grafting or Best Medical Treatment trial, TEVAR is beneficial for TBAD remodeling by repairing the primary entry tear and promoting thrombosis of the false lumen [4]. It is generally considered that false lumen thrombosis contributes to aortic remodeling [5]. The investigation of stent grafts in patients with TBAD trial revealed that 90.6% of patients benefit from completely thrombosed false lumen [6]. Conversely, patent and partially thrombosed false lumen are associated with negative outcomes [7,8].

Although several studies have investigated the risk factors for false lumen status in post-TEVAR aortas [9,10], the major determinants of false lumen status in pre-operative patients are still not clear. The purpose of the present study was to identify the factors that are associated with false lumen thrombosis in pre-operative TBAD patients.

## 2 Materials and methods

### 2.1 Study population

This was a single-center retrospective review of patients who presented to our center between January 2008 and December 2017. In this study, we enrolled consecutive patients diagnosed with TBAD by computed tomography angiography (CTA). Patients with type A aortic dissections, penetrating aortic ulcer, aortic intramural hematoma, and previous TEVAR were excluded. Outpatients were also excluded from this study.

### 2.2 Clinical variables

All data were obtained from the first examination of the patients’ hospitalization. Based on the false lumen status, the patients were divided into complete thrombosis, patent, and partial thrombosis groups. Complete thrombosis was defined as the absence of contrast media in the false lumen. Patent false lumen was defined as no thrombosis in the false lumen. Partial thrombosis was defined as the presence of both thrombosis and contrast media in the false lumen [11]. The false lumen status was assessed according to the CTA imaging.

Demographic, laboratory, and imaging data were retrospectively obtained. Demographic information, such as body mass index (BMI), age, and sex, was collected. In this study, we evaluated the major prehospital comorbidities that may affect TBAD patients. The prehospital comorbidities included smoking, coronary heart disease (CHD), renal impairment, chronic obstructive pulmonary disease (COPD), diabetes mellitus, stroke, hypertension, and peripheral arterial disease (PAD). CHD was defined as the presence of atherosclerotic plaques in the coronary arteries that lead to narrowing of the coronary arteries. Renal impairment was defined as an estimated glomerular filtration rate of less than 60 mL/min/1.73 m^2^ [12]. The main symptoms of the patients at disease onset were also recorded. The main symptoms included chest tightness, chest pain, stomach ache, lumbago, and backache.

We also recorded the laboratory data, including white blood cell count, red blood cell count, platelet count, hemoglobin, prothrombin time (PT), fibrinogen (FIB), thrombin time (TT), and activated partial thromboplastin time (APTT). We also recorded the blood pressure recorded at admission.

### 2.3 Measurements

**Figure 1:**
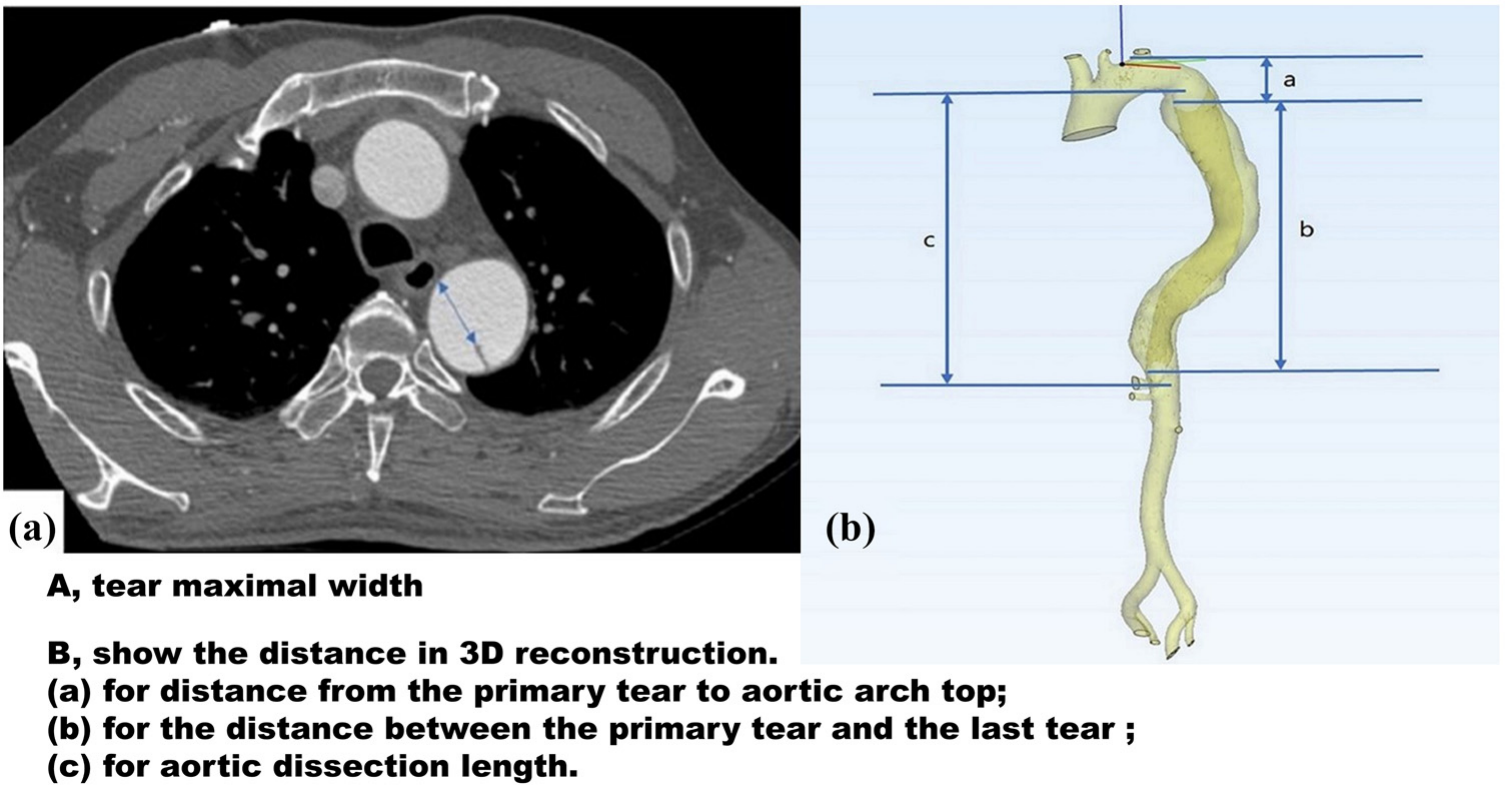
Aortic parameters.

We used CTA images to analyze the morphological characteristics of TBAD (shown in Figure 1). Arterial phase CTA images were used for all measurements. Two authors examined the CTA independently, and discrepancies were resolved by negotiation. We analyzed the sagittal, coronal, and transverse section images to evaluate whether the visceral arteries arise from the true lumen, false lumen, or both true and false lumens. The maximal width of the primary tear and last re-entrance tear were measured on the transverse section images. To evaluate the number of tears, we analyzed CTA images of the entire aorta. Since the false lumen was completely filled with the thrombus in the complete thrombosis models, the number and location of tears in this model could not be identified. Therefore, the comparison of tear characteristics did not involve the complete thrombosis group.

The distance from the primary tear to the top of the aortic arch was defined as the vertical distance from the maximal width plane of the primary tear to the top of the aortic arch. The distance between the primary tear and the last re-entrance tear was defined as the vertical distance between the maximal widths of the primary tear and the last re-entrance tear. The AD length was defined as the vertical distance from the highest point to the lowest point of the dissection.

The data were acquired from thin-slice and enhanced multidetector spiral CTA images performed at our center. Structural features of AD were clearly identified on all CTA images. Measurements were confirmed in three directions: coronal, sagittal, and cross-sectional. Three-dimensional (3D) reconstruction was also performed to characterize the morphological features of AD. Imaging data were saved in Digital Imaging and Communications in Medicine format and imported into MIMICS software (version 21.0; Materialise HQ, Leuven, Belgium) for 3D reconstruction and measurement.

### 2.4 Statistical analysis

According to the normality of data distribution, continuous data were presented as mean and standard deviation or median and interquartile range. Categorical data were reported as counts and percentages. Chi-square test was used to compare group differences for categorical variables. One-way analysis of variance and Student’s *t*-test were used to analyze the continuous variables. Kruskal–Wallis test was applied for non-parametric tests. Multivariable logistic regression models were developed to determine the predictors of thrombosis in the false lumen. Binary logistic analysis was used to determine the relationships between the tear parameters and false lumen thrombosis. In the primary comparison of baseline characteristics, factors with *p* values <0.05 were included in the model. *p* values <0.05 were considered statistically significant. The statistical analyses were performed using the SPSS 25.0 software package (IBM Corp., Armonk, NY, USA).


**Ethical approval:** This is a retrospective, observational study. The study was approved by the ethics committee of the First Affiliated Hospital of Guangxi Medical University, which waived the requirement for informed consent (No. 2022-KY-E-(206)).

## 3 Results

### 3.1 Clinical and demographic characteristics

During the study period of 10 years, 738 patients were diagnosed with AD. We excluded 388 patients with type A AD and 65 patients for whom data were not available. Finally, 285 patients were included in our analysis. The clinical and demographic characteristics of patients are summarized in Table 1. The median time from onset to first CTA was 96 h, and there was no statistical difference among the three groups. The participants had a mean age of 53 ± 11 years (range, 20–81), and 84% of participants were males. The most common comorbidity was hypertension (90%). In total, 51% of patients presented with chest pain. There were no significant differences in the symptoms between the three groups. We investigated the relationships between coagulation function, blood components, and false lumen thrombosis. Significant differences were present in the PT and FIB values between the three groups. Table S1 summarizes the demographic characteristics and laboratory parameters of the patent group versus the partial thrombosis group.

**Table 1:** Demographic characteristics and laboratory parameters of patients

Variables	Total (*n* = 285)	Patent (*n* = 115)	Partial thrombosis (*n* = 144)	Complete thrombosis (*n* = 26)	*p* value
Male	238 (83.5)	96 (83.5)	123 (85.4)	19 (73.1)	0.296
Age (years)	52.51 ± 11.33	50.44 ± 11.04	52.91 ± 11.32	59.42 ± 9.93	0.001
SBP (mmHg)	151.54 ± 28.20	151.76 ± 29.42	152.13 ± 28.34	147.35 ± 21.84	0.726
DBP (mmHg)	90.72 ± 44.83	89.56 ± 20.95	92.35 ± 59.94	86.77 ± 15.43	0.791
Time from onset to CTA (h)	96 (24,240)	96 (24,336)	120 (24,240)	72 (18,300)	0.771
BMI (kg/m²)	24.73 (22.37, 27.34)	24.39 (22.09, 27.34)	24.82 (22.59, 27.34)	24.52 ± 3.38	0.597
**Comorbidities**					
Hypertension	256 (89.8)	103 (89.6)	129 (89.6)	24 (92.3)	0.908
PAD	9 (3.2)	4 (3.5)	3 (2.1)	2 (7.7)	0.312
Diabetes	23 (8.1)	6 (5.2)	15 (10.4)	2 (7.7)	0.311
COPD	14 (4.9)	6 (5.2)	8 (5.6)	0 (0)	0.474
Smoking	103 (36.1)	42 (36.5)	52 (36.1)	9 (34.6)	0.983
Stroke	31 (10.9)	11 (9.6)	17 (11.8)	3 (11.5)	0.842
CHD	26 (9.1)	6 (5.2)	14 (9.7)	6 (23.1)	0.016
Renal impairment	32 (11.2)	20 (17.4)	12 (8.3)	0 (0)	0.012
**Symptom**					
Chest pain	144 (50.5)	61 (53.0)	71 (49.3)	12 (46.2)	0.750
Chest tightness	44 (15.4)	15 (13.0)	28 (19.4)	1 (3.8)	0.084
Stomach ache	67 (23.5)	23 (20)	36 (25)	8 (30.8)	0.422
Backache	46 (16.1)	22 (19.1)	19 (13.2)	5 (19.2)	0.393
Lumbago	22 (7.7)	9 (7.8)	13 (9.0)	0 (0)	0.283
**Laboratory parameters**					
PT (s)	11.96 ± 1.66	11.56 ± 1.62	12.24 ± 16.8	12.19 ± 1.31	0.003
APTT (s)	32.43 ± 6.29	32.03 ± 3.74	32.77 ± 8.06	32.41 ± 4.17	0.655
TT (s)	12.59 ± 5.14	11.88 ± 1.60	13.26 ± 7.07	12.14 ± 1.87	0.093
FIB (g/L)	5.68 ± 2.67	5.55 ± 1.64	5.45 ± 1.94	7.54 ± 6.58	0.001
WBC (×10^9^/L)	10.31 ± 4.14	9.95 ± 4.15	10.34 ± 4.26	11.77 ± 3.02	0.127
RBC (×10^12^/L)	4.22 ± 0.96	4.21 ± 1.01	4.26 ± 0.98	4.12 ± 0.64	0.779
HGB (g/L)	118.30 ± 21.35	115.70 ± 22.79	120.52 ± 21.07	117.51 ± 14.41	0.194
PLT (×10^9^/L)	245.52 ± 120.42	243.24 ± 97.36	241.17 ± 132.66	279.52 ± 140.11	0.318

Continuous data are presented as mean ± standard deviation or median (25th and 75th percentile). Categorical data are expressed as number (%). SBP, systolic blood pressure; DBP, diastolic blood pressure; BMI, body mass index; PAD, peripheral arterial disease; COPD, chronic obstructive pulmonary disease; CHD, coronary heart disease; PT, prothrombin time; APTT, activated partial thromboplastin time; TT, thrombin time; FIB, fibrinogen; WBC, white blood cell; RBC, red blood cell; HGB, hemoglobin; PLT, platelet.

### 3.2 Morphological measurements of AD

The morphological characteristics of the 285 patients are shown in Table 2. Tears were more common in the patent group than the partial thrombosis group (5 ± 2 vs 4 ± 2, respectively). The sizes of the primary and last tears in the patent group were 14 ± 8 and 8 ± 6 mm, respectively. Most visceral branches originated from the true lumen. There were significant differences in the distance from the primary tear to the top of the aortic arch between the patent false lumen and partial thrombosis groups (20[12–28] and 24[15–49] mm, respectively). The distance between the primary tear and the last re-entrance tear was 307 ± 106 mm in the patent false lumen group and 234 ± 140 mm in the partial thrombosis group (*p* < 0.05). Our analysis revealed that the AD length was significantly higher in the partial thrombosis group than in the complete thrombosis group 354 ± 92 vs 290 ± 101 mm, respectively). Table S2 summarizes the morphological characteristics of the patent group versus the partial thrombosis group.

**Table 2:** Morphological characteristics

Variables		Total (*n* = 285)	Patent (*n* = 115)	Partial thrombosis (*n* = 144)	Complete thrombosis (*n* = 26)	*p* value
Distance from primary tear to aortic arch top (mm)			19.75 (12.22,28.48)	24.1 (15.05,48.98)		0.000
Distance between primary tear and the last tear (mm)			306.85 ± 105.94	234.31 ± 139.50		0.000
Total number of tears			4.77 ± 2.08	4.10 ± 2.27		0.016
Primary tear size (mm)			13.51 ± 8.38	10.34 ± 7.78		0.002
The last tear size (mm)			7.61 ± 6.19	5.66 ± 3.84		0.002
Aortic dissection length (mm)		347.03 ± 98.02	350.71 ± 101.57	354.32 ± 91.89	290.46 ± 100.56	0.008
**Branches arose true or false lumen**						
Celiac trunk	FL	40 (14.0)	14 (12.2)	21 (14.6)	5 (19.2)	0.751
	TL	175 (61.4)	71 (61.7)	87 (60.4)	17 (65.4)	
	TL and FL	70 (24.6)	30 (26.1)	36 (25.0)	4 (15.4)	
SMA	FL	8 (2.8)	3 (2.6)	3 (2.1)	2 (7.7)	0.456
	TL	206 (72.3)	86 (74.8)	104 (72.2)	16 (61.5)	
	TL and FL	71 (24.9)	26 (22.6)	37 (25.7)	8 (30.8)	
Left renal artery	FL	55 (19.3)	22 (19.1)	26 (18.1)	7 (26.9)	0.191
	TL	197 (69.1)	75 (65.2)	107 (74.3)	15 (57.7)	
	TL and FL	33 (11.6)	18 (15.7)	11 (7.6)	4 (15.4)	
Right renal artery	FL	48 (16.8)	19 (16.5)	26 (18.1)	3 (11.5)	0.788
	TL	204 (71.6)	80 (69.6)	104 (72.2)	20 (76.9)	
	TL and FL	33 (11.6)	16 (13.9)	14 (9.7)	3 (11.5)	

SMA, superior mesenteric artery; TL, true lumen; FL, false lumen. Continuous data are presented as mean ± standard deviation or median (25th and 75th percentile). Categorical data are expressed as number (%).

### 3.3 Risk factor analysis

We found a total of 11 parameters with *p* values <0.05 in the univariate analysis. These parameters were selected for logistic regression analysis and included the age, presence of CHD and renal impairment, PT, FIB, total number of tears, size of the primary and last tears, distance from the primary tear to the top of the aortic arch, distance between the primary tear and the last re-entrance tear, and AD length.

Binary logistic regression analysis indicated that the factors for incomplete thrombosis in the false lumen were the size of primary tears (odds ratio [OR], 0.96; *p* = 0.03) and last tears (OR, 0.93; *p* = 0.04), and the total number of tears (OR, 0.87; *p* = 0.02) (shown in Table 3). According to the multivariate logistic regression analysis, age and AD length were associated with false lumen thrombosis (*p* < 0.05). Renal impairment tends to maintain the patency of the false lumen. The results are presented in Table 4.

**Table 3:** Relationship between five tear parameters and false lumen status in a total of 259 patients in the patent and partial thrombosis groups

Variables	OR	95% CI	*p* value
Distance from primary tear to aortic arch top	1.004	0.999–1.010	0.147
Distance between primary tear and the last tear	0.997	0.994–1.000	0.052
Total number of tears	0.87	0.776–0.976	0.017
Primary tear size	0.963	0.930–0.997	0.034
The last tear size	0.929	0.868–0.995	0.035

CI, confidence interval; OR, odds ratio.

**Table 4:** Relationship between six parameters and false lumen status in a total of 285 patients

A. Control group was complete thrombosis group						
Variables	Patent (*n* = 115)			Partial thrombosis (*n* = 144)		
	OR	95% CI	*p* value	OR	95% CI	*p* value
Age	0.951	0.912–0.991	0.016	0.970	0.932–1.009	0.127
PT	0.858	0.616–1.195	0.364	1.150	0.839–1.578	0.385
FIB	0.791	0.609–1.025	0.077	0.738	0.572–0.950	0.019
Aortic dissection length	1.005	1.000–1.009	0.036	1.006	1.001–1.010	0.008
CHD [contrast = 1a]	4.221	1.096–16.251	0.036	2.667	0.826–8.609	0.101
Renal impairment [contrast = 1b]	0 (0–0)	0.000–0.000	0.000	0 (0–0)	0.000–0.000	

B. Control group was partial thrombosis group						
Variables	Patent (*n* = 115)			Complete thrombosis (*n* = 26)		
	OR	95% CI	*p* value	OR	95% CI	*p* value
Age	0.980	0.957–1.004	0.107	1.031	0.991–1.073	0.127
PT	0.746	0.621–0.895	0.002	0.869	0.634–1.192	0.385
FIB	1.072	0.926–1.240	0.352	1.356	1.052–1.747	0.019
Aortic dissection length	0.999	0.996–1.002	0.455	0.994	0.990–0.999	0.008
CHD [contrast = 1a]	1.583	0.563–4.451	0.384	0.375	0.116–1.210	0.101
Renal impairment [contrast = 1b]	0.496	0.223–1.103	0.085	11932432.089	11932432.089–11932432.089	

CHD, coronary heart disease. PT, prothrombin time. FIB, fibrinogen. CI, confidence interval; OR, odds ratio. a, the patient with CHD. b, the patient with renal impairment.

## 4 Discussion

To evaluate the factors related to preoperative false lumen thrombosis, we analyzed the clinical features and morphological characteristics of AD in 285 patients. We found that TBAD patients who were older than 59 years or with AD lengths less than 290 mm were more likely to experience thrombosed false lumens. These findings suggest that TBAD patients older than 59 years of age or with AD lengths <290 mm may benefit from conservative treatment.

The multivariate logistic regression analysis showed that age was an influence factor of false lumen thrombosis. Larsen et al. [13] also found that the probability of complete thrombosis of the false lumen increased with age. Our findings are in line with those of Van Bogerijen et al. [14], who retrospectively analyzed 18 studies, and showed that age <60 years was a predictor of AD dilatation, while age ≥60 years was associated with a reduced risk of expansion. In contrast, Kitamura et al. [15] found that the age of the patients was significantly lower in the thrombosis group than the non-thrombotic group in TBAD after TEVAR. This discrepancy may originate from differences in study participants. Our participants had not undergone surgery, whereas those included by Kitamura et al. had undergone surgical treatment. Although age is associated with false lumen thrombosis in AD, the effects of age on thrombosis and the potential underlying mechanisms are not clear.

Our findings suggested that complete false lumen thrombosis was more prevalent in cases of shorter AD length. The AD length in the complete thrombosis group was significantly shorter (290 ± 101 mm) compared to the patent and partial thrombosis groups. Wang et al. [16] showed that the descending aorta was more prone to dilatation in patients with AD length ≥ descending aorta length. In a previous study, age was negatively correlated with AD length [17]. In this study, we found that the shortest length AD group was significantly older than the other groups.

Kotelis et al. [18] reported that TBAD with more than two tears is at risk for aortic dilatation. Tolenaar et al. [19] indicated that a decreased aortic growth rate suggested a location of the false lumen on the aortic outer curvature. Univariate and binary logistic regression analyses revealed that the size of the primary and last tears and the total number of tears are related to the false lumen status. The tear size directly affects the flow rate and pressure in the false lumen [20]. Higher false lumen flow velocity was more common with greater sizes of the entry and re-entry tears than smaller tears [21]. According to Li et al. [22], the presence of a primary tear larger than the last tear indicated that the pressure was higher in the false lumen than the true lumen, and was a risk factor for preoperative death. Ryzhakov et al. [23] also reported similar findings. Codner et al. [24] found that the distance from the primary tear to the left subclavian artery was a predictor of aortic growth. However, this study showed that the tear location was not a predictor of partial thrombosis. In the study by Codner et al. [24], the follow-up duration was 39 ± 34 months and 27 ± 27 months for the no-growth and growth groups, respectively. This study focused on a short period of time. We proposed that tear location has an insignificant impact on false lumen dilation during the short-term period. Furthermore, preoperative hemodynamics differ from postoperative hemodynamics in the AD model [25]. On the other hand, >2 tears could serve as a risk factor for false lumen expansion after TEVAR [26]. This study also showed that a greater number of tears were found in the patent group.

## 5 Strength and limitations

### 5.1 Strength

This study aimed to investigate the factors of false lumen thrombosis in 285 pre-operative TBAD patients by exploring three distinct dimensions: demographic factors, morphological characteristics, and blood parameter indices. We found that TBAD patients over the age of 59 years or with shorter aortic dissection lengths (<290 mm) showed a higher likelihood of experiencing thrombosed false lumens.

### 5.2 Limitations

First, this was a single-center retrospective study, which might have introduced bias in the data collection. Second, this study did not account for anatomical variants of the aorta. Third, this study focused on the false lumen status of preoperative patients, and the effect of false lumen status on prognosis still needs to be further explored.

## 6 Conclusions

TBAD patients older than 59 years or with AD lengths less than 290 mm were more likely to have a thrombosed false lumen.

## Abbreviations

3D: three-dimensional
AD: aortic dissection
APTT: activated partial thromboplastin time
BMI: body mass index
CHD: coronary heart disease
CI: confidence interval
COPD: chronic obstructive pulmonary disease
CTA: computed tomography angiography
FIB: fibrinogen
OR: odds ratio
PAD: peripheral arterial disease
PT: prothrombin time
TBAD: type B aortic dissection
TEVAR: thoracic endovascular aortic repair
TT: thrombin time

## Supplementary Material

Supplementary Table
