# Supplementary material for: Influencing factors of false lumen thrombosis in type B aortic dissection: A single-center retrospective study
Source: Open Med (Wars). 2025 May 7;20(1):20251179. doi: 10.1515/med-2025-1179 (PMC12086627; doi:10.1515/med-2025-1179)
Supplement: Supplementary Table [file med-2025-1179-sm.pdf]

Supplementary material

Table S1: Demographic characteristics and laboratory parameters in the patent and partial thrombosis groups

Variables	Total (*n* = 259)	Patent (*n* = 115)	Partial thrombosis (*n* = 144)	*P* value
Male	219(84.6)	96(83.5)	123(85.4)	0.668
Age(years)	51.81 ± 11.24	50.44 ± 11.04	52.91 ± 11.32	*0.079*
SBP(mm Hg)	151.96 ± 28.76	151.76 ± 29.42	152.13 ± 28.34	0.917
DBP(mm Hg)	91.11 ± 46.77	89.56 ± 20.95	92.35 ± 59.94	0.633
Time from onset to CTA(h)	96(24,240)	96(24,336)	120(24,240)	0.547
BMI (kg/m^2)	24.69(22.44, 27.34)	24.39(22.09, 27.34)	24.82(22.59, 27.34)	0.312
**Comorbidities**				
Hypertension	232(89.6)	103(89.6)	129(89.6)	0.996
PAD	7(2.7)	4(3.5)	3(2.1)	0.762
Diabetes	21(8.1)	6(5.2)	15(10.4)	0.128
COPD	14(5.4)	6(5.2)	8(5.6)	0.905
Smoking	94(36.3)	42(36.5)	52(36.1)	0.946
Stroke	28(10.8)	11(9.6)	17(11.8)	0.564
CHD	20(7.7)	6(5.2)	14(9.7)	*0.177*
Renal impairment	32(12.4)	20(17.4)	12(8.3)	*0.028*
**Symptom**				
Chest pain	132(51.0)	61(53.0)	71(49.3)	0.550
Chest tightness	43(16.6)	15(13.0)	28(19.4)	0.169
Stomach ache	59(22.8)	23(20)	36(25)	0.340
Backache	41(15.8)	22(19.1)	19(13.2)	0.193
Lumbago	22(8.5)	9(7.8)	13(9.0)	0.730
**Laboratory parameters**				
PT(s)	11.94 ± 1.69	11.56 ± 1.62	12.24 ± 16.8	0.001
APTT(s)	32.44 ± 6.48	32.03 ± 3.74	32.77 ± 8.06	0.371
TT(s)	12.63 ± 5.37	11.88 ± 1.60	13.26 ± 7.07	0.041
FIB(g/L)	5.49 ± 1.81	5.55 ± 1.64	5.45 ± 1.94	0.676
WBC(×10^9/L)	10.17 ± 4.21	9.95 ± 4.15	10.34 ± 4.26	0.462
RBC(×10^{12}/L)	4.23(3.69, 4.74)	4.21 ± 1.01	4.26 ± 0.98	0.379
HGB(g/L)	118.37 ± 21.94	115.70 ± 22.79	120.52 ± 21.07	0.079
PLT(×10^9/L)	242.09 ± 118.03	243.24 ± 97.36	241.17 ± 132.66	0.889

Continuous data are presented as mean ± standard deviation or median (25th and 75th percentile). Categoric data are expressed as number (%). SBP, systolic blood pressure. DBP, diastolic blood pressure. BMI, body mass index. PAD, peripheral arterial disease. COPD, chronic obstructive pulmonary disease. CHD, coronary heart disease. PT, prothrombin time. APTT, activated partial thromboplastin time. TT, thrombin time. FIB, fibrinogen. WBC, white blood cell. RBC, red blood cell. HGB, hemoglobin. PLT, platelet.

Table S2: Morphological characteristics in the Patent and Partial Thrombosis Groups

Variables		Total (n = 259)	Patent (n = 115)	Partial thrombosis (n = 144)	p value
Distance from primary tear to aortic arch top, mm		21.8(14.07, 33.33)	19.75(12.22, 28.48)	24.1(15.05, 48.98)	0.000
Distance between primary tear and the last tear, mm		266.52 ± 130.57	306.85 ± 105.94	234.31 ± 139.50	0.000
Total number of tears		4.40 ± 2.20	4.77 ± 2.08	4.10 ± 2.27	0.016
Primary tear size, mm		11.74 ± 8.19	13.51 ± 8.38	10.34 ± 7.78	0.002
The last tear size, mm		6.53 ± 5.11	7.61 ± 6.19	5.66 ± 3.84	0.002
Aortic dissection length, mm		352.71 ± 96.14	350.71 ± 101.57	354.32 ± 91.89	0.765
**Branches arose true or false lumen**					
Celiac trunk	FL	35(13.5)	14(12.2)	21(14.6)	0.851
	TL	158(61.0)	71(61.7)	87(60.4)	
	TL and FL	66(25.5)	30(26.1)	36(25.0)	
SMA	FL	6(2.3)	3(2.6)	3(2.1)	0.825
	TL	190(73.4)	86(74.8)	104(72.2)	
	TL and FL	63(24.3)	26(22.6)	37(25.7)	
Left renal artery	FL	48(18.5)	22(19.1)	26(18.1)	0.108
	TL	182(70.3)	75(65.2)	107(74.3)	
	TL and FL	29(11.2)	18(15.7)	11(7.6)	
Right renal artery	FL	45(17.4)	19(16.5)	26(18.1)	0.571
	TL	184(71.0)	80(69.6)	104(72.2)	
	TL and FL	30(11.6)	16(13.9)	14(9.7)	

SMA, superior mesenteric artery. TL, true lumen. FL, false lumen. Continuous data are presented as mean ± standard deviation or median (25th and 75th percentile). Categoric data are expressed as number (%).
